# Clinical Applications of Multimodal Artificial Intelligence in Otolaryngology: A State‐of‐the‐Art Review

**DOI:** 10.1002/ohn.70285

**Published:** 2026-05-12

**Authors:** Ying Jie Li, Flora Su, Norbert Banyi, Philip Edgcumbe, Andrew Thamboo, Ameen Amanian

**Affiliations:** ^1^ Faculty of Medicine University of British Columbia Vancouver British Columbia Canada; ^2^ Department of Science University of British Columbia Vancouver British Columbia Canada; ^3^ Division of Otolaryngology–Head and Neck Surgery, Department of Surgery University of British Columbia Vancouver British Columbia Canada; ^4^ Department of Radiology University of British Columbia Vancouver British Columbia Canada

**Keywords:** artificial intelligence, deep learning, multimodal, natural language processing

## Abstract

**Objective:**

Artificial intelligence (AI) has advanced to simultaneously process visual, auditory, and textual inputs, providing users with “multimodal” AI. Given the clinical integration potential of these tools, otolaryngologists must stay informed. This study reviews current literature on applications of multimodal AI in otolaryngology.

**Data Sources:**

The MEDLINE, EMBASE, SCOPUS, Cochrane Library, Web of Science, and CINAHL databases.

**Review Methods:**

Databases were searched from the date of inception to March 4, 2025, following Preferred Reporting Items for Systematic Reviews and Meta‐analyses extension for scoping reviews (PRISMA‐ScR) guidelines. Studies on any application of multimodal AI in otolaryngology were included.

**Conclusions:**

Forty‐four studies were included, with 55% (24/44) published in 2024 and 18% (8/44) in 2025. Image and text were the most commonly combined modalities (80%, 35/44), with emerging combinations including video with vector data (2%,1/44) and omics with text and/or image (14%, 6/44). Head and neck cancer was the most common subspecialty of focus (75%, 33/44), followed by general ear, nose, and throat (ENT) (11%, 5/44). All studies applied the models for clinical education (9%, 4/44) or decision support (91%, 40/44), assessing performance in areas such as board‐style examination performance (accuracy: 37%‐86%) or disease classification and prognostication (area under the receiver operating characteristic curve [AUC] 0.65‐0.96). However, most studies were limited to small, single‐institution samples and lacked prospective validation. Model error, data set bias, and language limitations underscore the need for further refinement.

**Implications for Practice:**

The application of multimodal large language models (LLMs) in otolaryngology is rapidly expanding. Clinicians must understand both the capabilities and limitations of these systems. Rigorous validation and ethical oversight will be essential to ensure the safe, equitable, and effective adoption in otolaryngologic care.

Artificial intelligence (AI) has demonstrated transformative potential across diverse domains of medicine, showcasing applications in diagnostics, prognostication, risk stratification, and workflow optimization.^1^, ^2^, ^3^ Among the most notable recent advancements are large language models (LLMs) under natural language processing, trained on vast textual corpora to perform increasingly sophisticated tasks. Tools such as generative pre‐trained transformers (GPTs), particularly OpenAI's ChatGPT, have garnered significant attention from both clinicians and the public. These developments have catalyzed further public and professional discourse on the utility of LLMs in clinical practice.^2^, ^4^

As AI systems evolve, multimodal AI has emerged as the next frontier, enabling simultaneous integration of diverse input modalities including text, image, audio, and video.^5^, ^6^ This allows for more contextualized outputs that mirror the integrative reasoning processes of clinicians.^1^ By synthesizing heterogeneous data streams such as medical imaging, laboratory results, clinical notes, and voice recordings, LLMs may support clinical decision‐making in ways that surpass unimodal systems. Already, multimodal AI has demonstrated promise in disease detection, risk prediction, differential diagnosis generation, and treatment planning across multiple specialties.^7^

The pace of innovation has accelerated in recent years. Since the initial launch of ChatGPT in 2022, the release of GPT‐4 Vision and GPT‐4o have signaled a rapid proliferation of multimodal LLMs, with forecasts estimating thousands of distinct multimodal models in use by 2025.^5^ Notably, 2025 open‐source models such as DeepSeek‐VL and Janus‐Pro7B have outperformed existing commercial counterparts including DALL‐E3 and TokenFlow‐XL in benchmark tasks, highlighting the increasing sophistication and accessibility of these systems.^8^ In parallel, the global AI healthcare market has surged by billions in just the past 3 years,^9^ with AI contributing an estimated $400 billion to the US economy as of 2024.^10^

As these models move from research labs into clinical environments, it is crucial to understand their implications for medical specialties, including otolaryngology–head and neck surgery (OHNS), where multimodal data are already a routine part of practice. Otolaryngologists routinely use videolaryngoscopy, audiometry, voice recordings, and radiologic imaging alongside electronic health records (EHRs), creating rich multimodal data sets well‐suited for use by AI technologies.^11^ Although still in its infancy, multimodal AI has promising potential for tasks such as image segmentation,^12^ interpretation of endoscopic or radiologic imaging for malignancy detection, disease diagnoses and characterization,^13^ or surgical planning.^14^ Beyond diagnostics, AI integration could act as a helpful adjunct for clinicians and trainees to enhance efficiency and improve patient outcomes across the diverse responsibilities of clinical care, education, research, and administrative domains.^15^

Given the pace of innovation and the multimodal nature of otolaryngologic data, it is imperative for clinicians and researchers in the field to stay informed and engaged. This state‐of‐the‐art review aims to examine the current literature on the use of multimodal AI in otolaryngology and identify existing applications, challenges, and areas for future development.

## Methods

This review was conducted following the Preferred Reporting Items for Systematic Reviews and Meta‐analyses extension for scoping reviews (PRISMA‐ScR) checklist and the methodology was published on Open Science Framework (doi:10.17605/OSF.IO/S65VH).^16^

### Search Strategy

To identify relevant articles, the MEDLINE, EMBASE, SCOPUS, Cochrane Library, Web of Science, and CINAHL databases were searched from the date of inception to March 4, 2025.

The search was developed in collaboration with an institutional librarian. Subject headings and relevant keywords were combined with Boolean operators where appropriate (exact search strategy, Supplemental Table S1, available online). The search was modified for syntax across databases as necessary.

### Study Selection

Results from all databases were uploaded into the Covidence systematic review software (Veritas Health Innovation).^17^ Duplicates were automatically removed by the program. All original full‐text articles centered on the application of multimodal AI in otolaryngology were included. Articles were excluded if they were non‐English or lacked abstract and/or full text access. Study titles and abstracts were initially screened for inclusion by two independent reviewers (Y.J.L., F.S.), with discrepancies resolved by N.B. Y.J.L and F.S. then screened the full text of all short‐listed studies. Disagreements were settled through consensus by all three reviewers.

### Data Extraction

From the included full‐text articles, data were extracted in duplicate by Y.J.L. and F.S. following a predetermined template that included study type, level of evidence, publication year, country of senior author, objective, AI model, input modalities, application, OHNS subspecialty, sample size, methodology, and performance outcomes. Discrepancies were settled through consensus.

## Results

**Figure 1:**
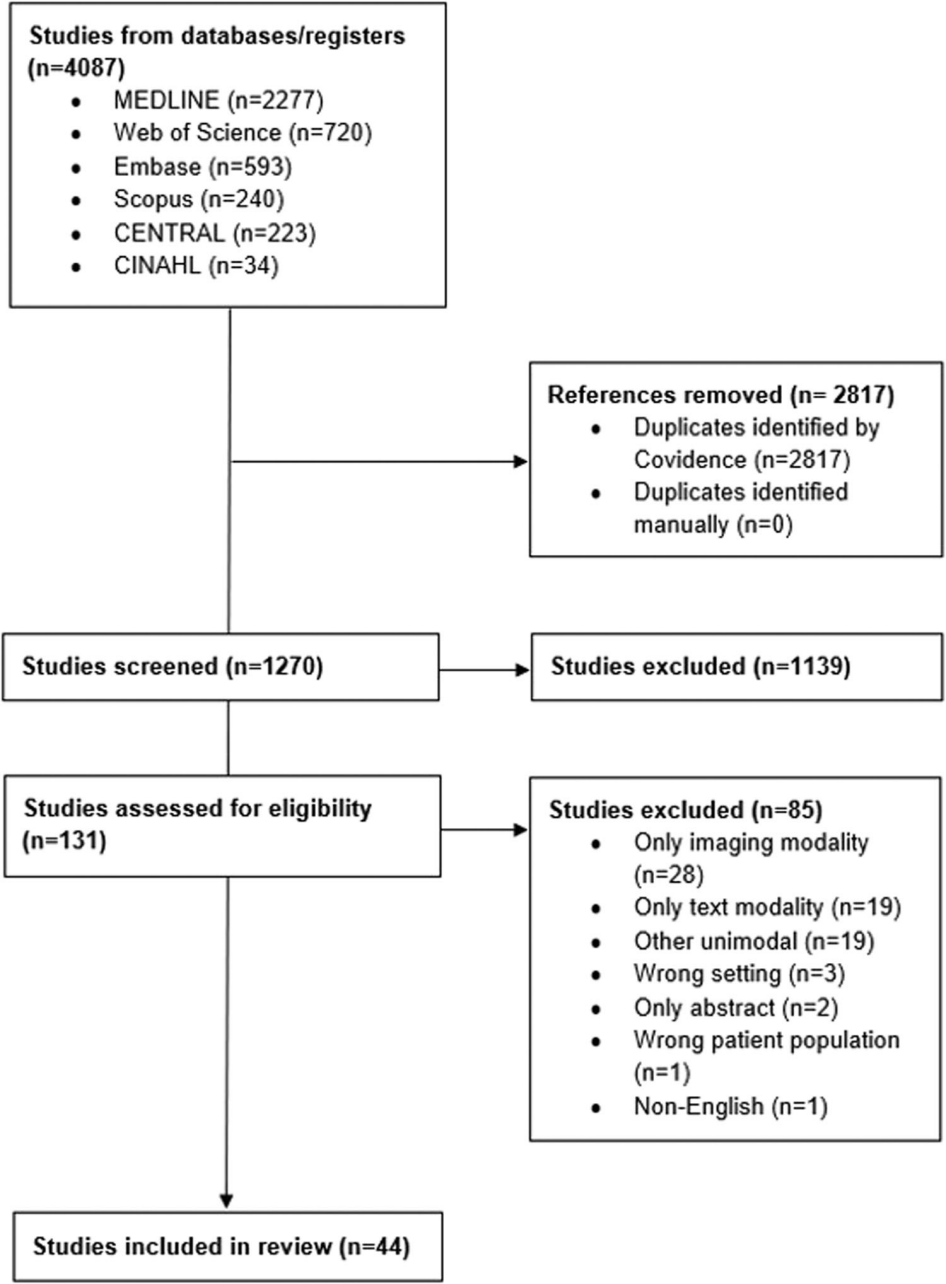
Preferred Reporting Items for Systematic Reviews and Meta‐analyses (PRISMA) flow diagram of study selection. Adapted from the PRISMA summarizing the number of studies identified, screened, and included along with the reasons for exclusion at each stage.

A total of 4087 studies were initially identified, and 2817 duplicates were removed. Following title and abstract screening, 131 articles were assessed in full, and 44 studies met the inclusion criteria (Figure 1).

### Study Characteristics

**Figure 2:**
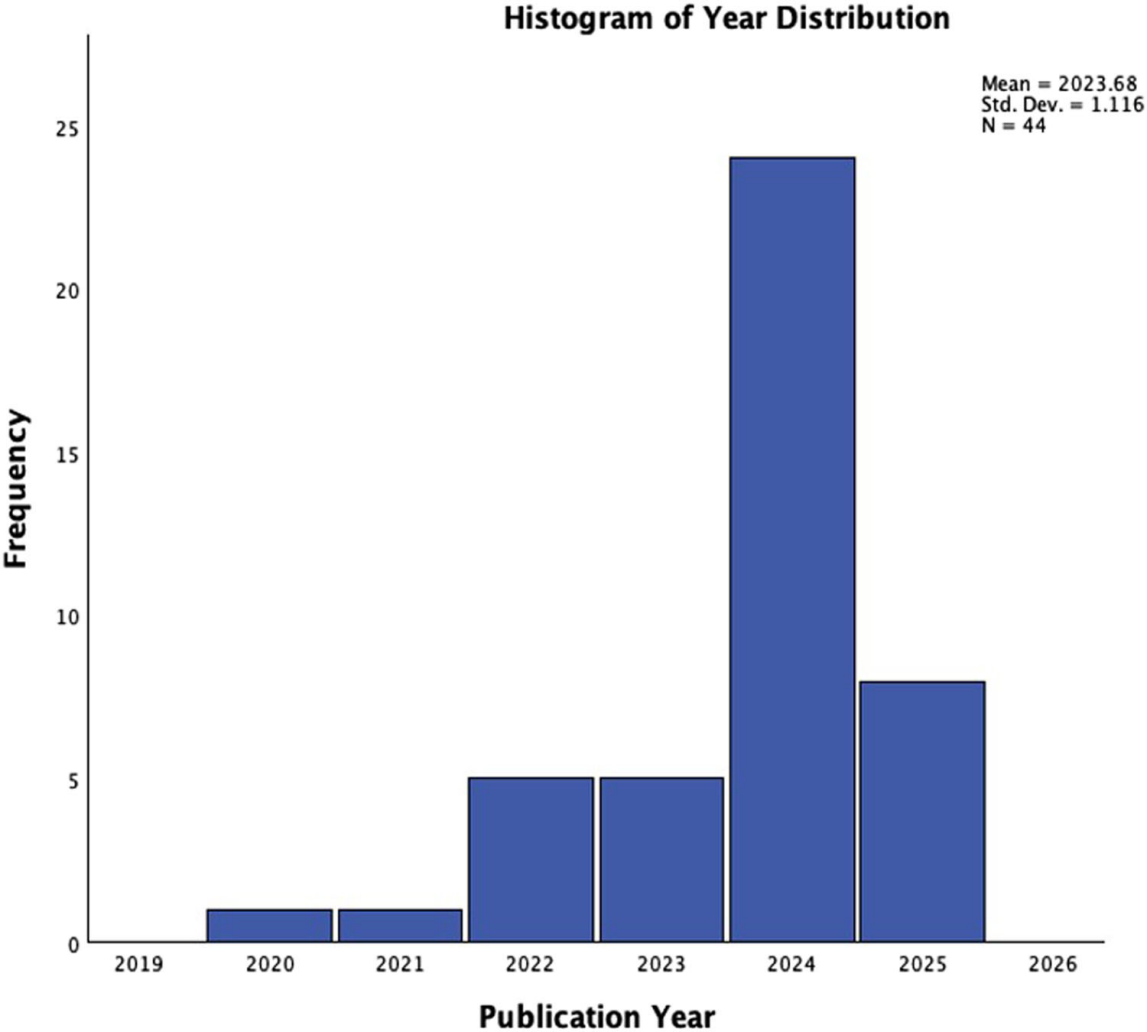
Publication year distribution of included studies. Histogram displaying the distribution of included studies by publication year, with a bin width of 1 year.

The median publication year was 2024 (range 2020‐2025) with 55% of all included studies being published in 2024 (Figure 2). The second leading year for publication was 2025 (18%). Most studies were published from China (n = 21, 47.7%), followed by United States (n = 11, 25%, Table 1). Other countries of publication included Canada, Germany, Japan, and Korea (each n = 2, 4.5%). All 44 studies explored clinical application for multimodal AI, predominantly in head and neck oncology (n = 33, 75%), followed by general otolaryngology (n = 5, 11%, Table 2). The remaining studies spanned subspecialties including rhinology, neurotology, laryngology, and otology. A detailed summary of model architectures and input features for each study is provided in Supplemental Table S2, available online. The study methods and outcomes are reported in Supplemental Table S3, available online.

**Table 1:** Number of Studies by Country of Senior Author^a^

Country	Number of studies, n (%)
China	21 (47.7)
United States	11 (25.0)
Canada	2 (4.5)
Germany	2 (4.5)
Japan	2 (4.5)
Korea	2 (4.5)
France	1 (2.3)
India	1 (2.3)
The Netherlands	1 (2.3)
Turkey	1 (2.3)

^a^ Distribution of included studies according to the country affiliation of the senior author.

**Table 2:** Number of Studies by Ear, Nose, and Throat (ENT) Subspecialty^a^

Subspecialty	Number of studies, n (%)
Head and neck oncology	33 (75.0)
General ENT	5 (11.4)
Rhinology	2 (4.5)
Neurotology	2 (4.5)
Laryngology	1 (2.3)
Otology	1 (2.3)

^a^ Distribution of included studies by otolaryngology subspecialty focus.

Due to the heterogeneity in study design, objectives, and outcome measures, no meta‐analysis was performed. All studies were classified as performance evaluation studies and could not be graded using the Oxford Levels of Evidence.

## Discussion

To the authors' knowledge, this review presents the most up‐to‐date comprehensive review of multimodal AI applications in the field of otolaryngology.

### AI Models and Input Modalities

Across the included studies, multimodal AI systems fell into two broad categories. The first consisted of publicly available or commercial LLMs (eg, LLaVA or ChatGPT), which are pre‐trained on large, publicly available data sets (eg, books, websites, scientific journal articles, and discussion forums).^18^ LLMs were originally only capable of text‐based tasks but have since evolved to accept and generate multiple input and output modalities. The second category comprised custom‐developed models, which were built and trained on institution and subject‐specific data sets. These models were designed with the capability to process and integrate multiple modalities of data using a customized architecture. Often, machine learning techniques such as convolutional neural networks (CNNs), support vector machines (SVMs), ResNet50, or gradient boosting were employed.

Fifteen studies utilized commercial LLMs including ChatGPT (versions 3.5, 4, 4Vision, 4o), Claude (3‐Opus, 3.5‐Sonnet), Gemini (1.5‐Pro), and Bing Co‐Pilot. The remaining studies used open‐source models (eg, LLaVA‐Med, InternVL) or custom‐built architectures. Studies either developed new models tailored to ENT problems or assessed the performance of existing LLMs in ENT‐specific scenarios.

**Figure 3:**
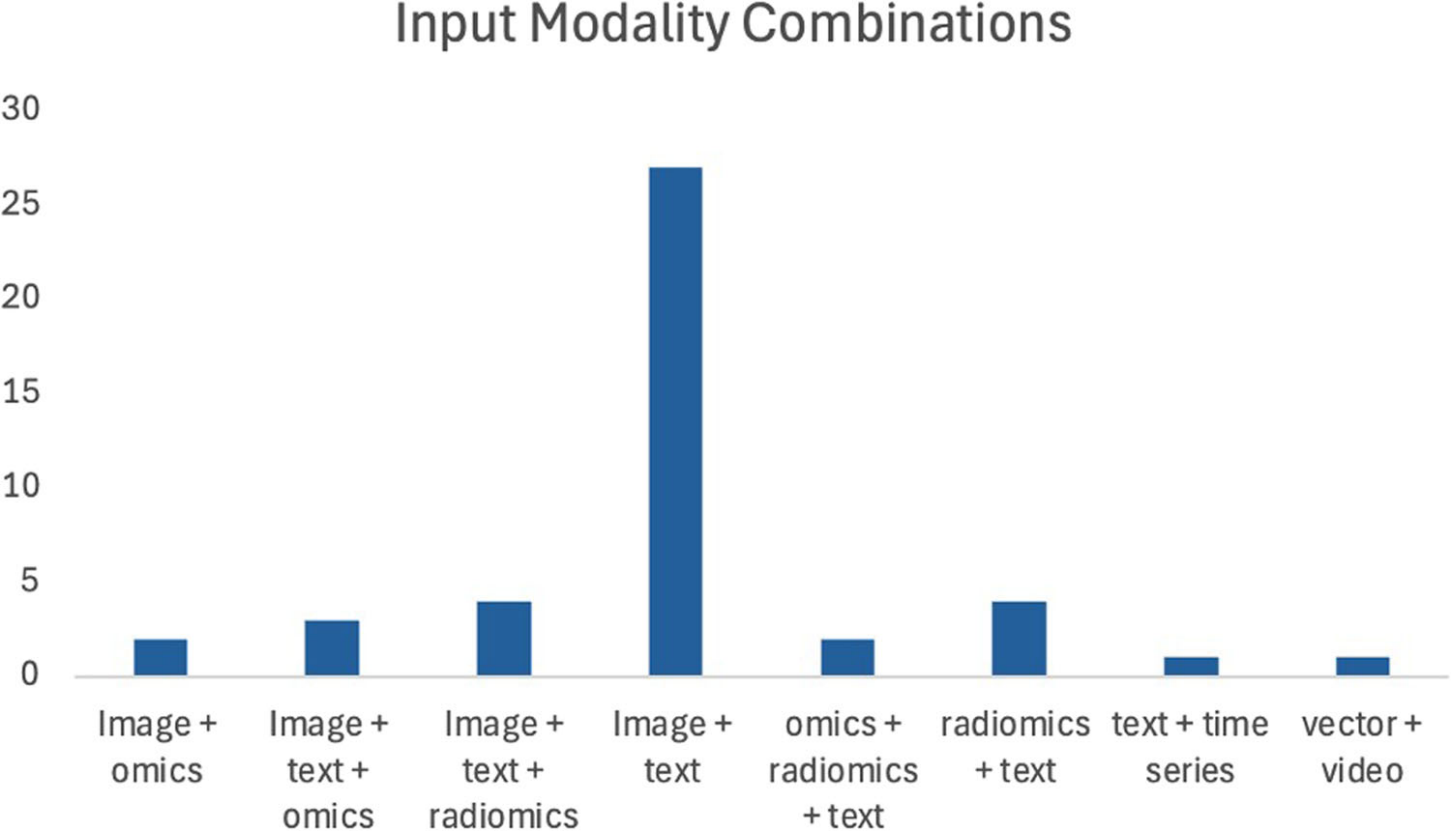
Number of studies by input modality combination. Bar graph summarizing the frequency of each unique modality combination utilized in the included studies.

Studies included data such as clinicodemographic information, imaging studies (magnetic resonance imaging [MRI], positron emission tomography [PET], computed tomography [CT], ultrasound [US], histopathological sections, and otoscopic images), omics data (radiomics, pathomics, and genomics), lab results (thyroid function, serum eosinophil count), and eye movement videos. Most studies combined two modalities, with image and text being the most frequent pairing (35/44, Figure 3).

### Clinical Applications

1. Clinical education
  The clinical acumen of medical trainees is often assessed through multiple‐choice exams, which aim to simulate the integration of knowledge and judgment required in real‐world decision‐making. To aid trainees in their study, many commercial platforms have developed question banks containing licensing exam‐style questions, incorporating both text and image‐based clinical scenarios.
  Four studies evaluated publicly available LLMs' capability of answering otolaryngology board‐style questions.^19^, ^20^, ^21^, ^22^ All studies drew questions verbatim from established question banks, and tasked LLMs to select the correct answer and provide an explanation for their selection.
  Across all models, accuracy for English inputs ranged from 37% to 86%, with the highest question response accuracy achieved by ChatGPT‐4. In fact, ChatGPT‐4 and Gemini's test‐taking performance across all question stems from various subspecialty sections in question bank‐generated exams was found to be comparable to junior residents, but could not compete with senior residents (accuracy 54.75% vs 75.5%, *P* < .001).^22^
  Interestingly, ChatGPT‐4's performance was significantly better when prompted with both text and image (41.3%) compared to text alone (30.4%, *P* = .02). For example, in questions which asked for the most likely diagnosis, ChatGPT‐4 was able to correctly identify diagnostic features such as overlapping nuclei, ground glass appearance, nuclear grooves, and occasional intranuclear inclusions, in the histologic image accompanying the question stem to assist in the correct selection of papillary thyroid carcinoma (PTC) for the answer.^21^ These findings highlight the potential value of multimodal integration in enhancing clinical reasoning tasks. However, the need for further refinement remains evident, as the highest image interpretation accuracy achieved by the LLMs was 51.5% for radiologic images, decreasing to 41.7% for histopathological images and further declining to 29.2% for physiologic data.^19^, ^21^
  Another factor influencing LMM test‐taking performance was the subspecialty topic. Terwilliger et al (2024) noted that the LLMs performed better on allergy and immunology questions. They postulated this to be due to the concrete and fact‐based nature of the questions, whereas other topics may require more nuanced or multi‐step reasoning.^21^ In some instances, for particularly complex questions or images, the LLM returned error messages such as “not enough information.” While some studies excluded these instances, others categorized them as “non‐response.” Thus, while LLMs demonstrate strong capabilities in pattern recognition and factual recall, their ability to perform higher‐order clinical reasoning remains limited. Although the memorization of foundational knowledge is essential in medical training, the development of true clinical competence relies heavily on the integration of contextual judgment, diagnostic reasoning, and real‐time decision‐making. As such, LLMs may be helpful to supplement study tools, particularly for early‐stage learners, but are not yet reliable substitutes for explaining expert clinical reasoning.
  The influence of language on model performance must also be considered. A technical report on GPT‐4 highlighted a 15% drop in language understanding between high‐resource languages like English and German compared to low‐resource languages such as Thai or Telugu.^22^
  One study specifically investigated ChatGPT‐4V's ability to answer board‐style questions from Japanese otolaryngology board examination questions. When Japanese text prompts were translated to English, the accuracy significantly rose (24.7% to 47.3%, *P* < .001) and the non‐response rate dropped (46.3% to 2.7%, *P* < .001).^19^ ChatGPT‐4's twofold increase in response accuracy when identical question stems were translated from Japanese to English, despite the unchanged underlying clinical reasoning task, highlights the model's reliance on its training language. These findings emphasize the need for multilingual model optimization to ensure equitable clinical utility across diverse patient and trainee populations.
2. Treatment planning and evaluation
  Eleven studies applied custom‐developed multimodal AI architectures, such as those developed on vision transformer backbones with LLM integration for text‐rich feature extraction,^23^, ^24^ to support radiation treatment planning and to predict treatment response or risk of complication following radiotherapy for head and neck cancer patients.^23^, ^24^, ^25^, ^26^, ^27^, ^28^, ^29^, ^30^, ^31^, ^32^, ^33^
  In head and neck oncology, radiation therapy is a common treatment modality, requiring precise delineation of target volumes and organs at risk (OAR) on imaging.^28^ Current image segmentation methods are laborious, time‐intensive, and sensitive to inter‐rater variation.^24^ To streamline and standardize this process, several studies have developed multimodal AI systems integrating image and textual clinical data. One example representative of this AI architecture is a vision language model, capable of feature extraction from 3D images (eg, T2‐weighted MRI), integrated with ChatGPT and PubMed BERT to extract and transform tumor‐related details from clinical notes into word vectors for target volume delineation.^23^ On public head and neck cancer and oropharyngeal carcinoma data sets, these models demonstrated Dice similarity coefficients (DSCs) between 0.76 and 0.81 and 95th percentile Hausdorff distances (HD95) between 7.82 and 9.86 mm for target volume delineation.^23^, ^24^ At one institution, a GPT‐4Vision (GPT‐4V)–based model “GPT‐RadPlan” provided more accurate plans for 75% of head and neck cases, reducing mean dose by 10% to 15% for OAR.^28^ Another team utilized a model incorporating eXtreme Gradient Boosting to risk stratify pre‐treatment nasopharyngeal carcinoma patients using deep learning MRI features and clinical information to determine whether chemo should be added to radiotherapy treatment, achieving a concordance index of 0.804 on the external validation cohort.^25^ Although these models were found to outperform existing state‐of‐the‐art models, and either outperformed or matched clinical plans,^23^, ^28^ the models had conflicting dosimetric priorities, such as balancing coverage of planning target volumes (PTVs) and sparing critical OARs. Future approaches could use prompt engineering methods that adjust the importance of different clinical goals in real time, helping to find the best possible balance between them.^28^ Progress in this field has the potential to streamline clinical workflows, boost efficiency, and reduce patient wait times, factors that collectively support better patient outcomes.^34^, ^35^
  Beyond treatment planning, some models applied multimodal AI to predict response to neoadjuvant chemoimmunotherapy or radiotherapy in esophageal and nasopharyngeal cancer patients. These models integrated textual clinical data with deep learning and radiomic features from CT or multi‐sequence MRI and functional magnetic resonance imaging (fMRI). Pathomics from whole slide images was included in the CT study. They demonstrated area under the receiver operating characteristic curve (AUC) values ranging from 0.89 to 0.96.^26^, ^33^ Though the predictive performance appears strong, the studies were limited by small, retrospective, and single‐institution samples, factors which reduce generalizability. One study further identified an underrepresentation of females and unequal distribution between squamous cell carcinoma (SCC) and adenocarcinoma in their cohort,^33^ factors which highlight the need for broader validation as these groups may respond differently to therapy.
  Further, this emphasizes the risk of biased model outputs when training data lacks demographic and clinical diversity. LLMs are inherently dependent on the data they are trained on, and biased or non‐representative data sets can propagate inaccuracies, reinforce stereotypes, and produce misleading clinical inferences. This distributional fallacy is not confined to institution‐specific head and neck data sets but extends across OHNS. For example, in an analysis of over 12,000 participants across US‐based prospective chronic rhinosinusitis (CRS) studies, racial, ethnic, and gender minorities were significantly underrepresented relative to their proportions in the general population.^36^, ^37^ Without deliberate efforts to ensure inclusivity and transparency in data set composition, unsupervised or poorly validated AI models may exacerbate existing disparities in care.^38^
  Other models looked at treatment complications such as radiation‐induced hypothyroidism, esophageal fistula formation, re‐irradiation necrosis, and the need for a feeding tube during radiation therapy. These models commonly integrated clinical features, dose‐volume histograms, and imaging‐derived radiomics or dosiomics using machine learning methods such as least absolute shrinkage and selection operator (LASSO) regression, eXtreme Gradient Boosting, random forests, CNNs, amongst other conventional classifiers. These studies found AUC values from 0.75 to 0.936,^27^, ^30^, ^31^, ^32^ demonstrating reasonable utility for risk stratification to reduce unnecessary treatment. However, further external validation is required prior to clinical deployment as these studies were limited to single‐center data prone to overfitting or bias to institutional data.
3. Disease classification
  Eleven studies investigated the utility of LLMs for disease classification.^39^, ^40^, ^41^, ^42^, ^43^, ^44^, ^45^, ^46^, ^47^, ^48^, ^49^
  Starting broadly, one study simulated diagnostic reasoning by prompting ChatGPT to generate a list of differential diagnoses given digital pathology imaging and clinical summaries for ameloblastoma, SCC, mucoepidermoid carcinoma, and pleomorphic adenoma. ChatGPT was able to include the correct diagnosis in 100% of the differential lists, ranked it first in 49.5% of cases, and within the top two in 83%.^41^ While promising, the model selected from pre‐defined options within a limited sample size, and only achieved 50% accuracy in the most likely differential. This approach may hold more utility in resource‐limited settings where access to specialists is limited, or as a tool for early triaging of complex cases. Possibly, by increasing physician awareness and diagnostic confidence, delays in treatment or referrals will be reduced.^34^, ^35^
  For diagnosing conditions including middle ear disease, oropharyngeal and laryngeal malignancies, accuracies ranged from 41.1% to 82.1% across open source and commercial models.^39^, ^42^, ^48^, ^49^ The highest accuracy was demonstrated by ChatGPT‐4V on diagnosing middle ear diseases using patient clinical data and otoscopic images of the tympanic membrane. Its performance surpassed certified pediatricians (70.6%) but still fell short of otolaryngologists (95%).^45^ In identifying oropharyngeal malignancy, ChatGPT‐4V demonstrated 71.2% accuracy based on confocal laser endomicroscopy images and clinical text, while ChatGPT‐4o achieved 64.2% accuracy based on text and images from case reports.^42^, ^48^ Despite this promising performance, ChatGPT‐4 exhibited only 27.5% accuracy in analyzing videolaryngostroboscopy images for diagnosing laryngeal conditions and indicated extraneous examinations 2× more often than practitioners (153 v. 63 extraneous tests ordered).^49^ Further, ChatGPT‐4o incorrectly diagnosed 14.2% of oropharyngeal malignancy cases.^42^
  AI models were also evaluated on the classification of thyroid nodules as benign or malignant using various input combinations including US images, hematoxylin and eosin (H&E)–stained histopathology slides, radiomics features, and clinical data. In these tasks, LLMs were prompted to evaluate the images based on composition, echogenicity, homogeneity, shape, margins, and presence or absence of calcifications and determine malignancy risk. Although they achieved AUCs from 0.79 to 0.97,^40^, ^43^, ^44^, ^46^ agreement with pathological results was poor (kappa 0.116).^43^ Further, the rate of unnecessary biopsy was high (41%‐43%), compared to only 12.1% for junior radiologists who were asked to evaluate the same thyroid US and clinical excerpts based on ACR TI‐RADS guidelines.^43^
  Beyond text and images, one study developed a multimodal deep learning model for the diagnosis of benign paroxysmal positional vertigo (BPPV), using eye movement video and head position vector information. It achieved an 81.7% accuracy.^47^
  Overall, LLMs and multimodal AI architectures hold promise as diagnostic aids. Although diagnostic accuracy varied across studies, multimodal models outperformed their single modality comparators within the included studies.^44^, ^45^, ^46^ This showcases the value of integrating diverse inputs to enhance predictive performance. Of commercial models, ChatGPT‐4o and Claude 3.5 Sonnet exhibited the highest diagnostic accuracy, greater concordance with subject experts, and a notable reduction in unnecessary tests.^39^, ^40^, ^42^ However, their performance remained highly variable across clinical tasks and subspecialties. Even at their best, these systems did not match the diagnostic accuracy of board‐certified otolaryngologists, particularly in subspecialties like laryngology. The production of incorrect diagnoses or recommendation of excessive investigations underscores their limitations in high‐stakes diagnostic contexts. The consequences of diagnostic errors can be profound, and the onus of responsibility ultimately lies with the physician.^38^ Particularly due to the “black box” nature of many commercial LLMs such as ChatGPT, the decision‐making process can be hard to re‐trace and document.
  Importantly, the heavy cognitive load, task complexity, and time limitation in the clinical workplace increase risk for automation bias, defined as the human propensity to emphasize machine outputs.^50^, ^51^ This may lead to the disregard for potential errors or contradicting information, and has been substantiated in many contexts.^52^, ^53^
  Unlike AI scribes, which are primarily intended to reduce documentation burden, helping to alleviate cognitive load and after‐hours work by nearly one‐third,^54^ AI systems in this review engage with higher‐order clinical decision‐making tasks. While emerging AI scribes may further improve workflow efficiency and physician well‐being, decision‐support AI operates in a different risk domain. In such contexts, clinicians must remain critically engaged as these systems remain fallible and should serve only as adjuncts rather than replacements for clinical expertise.
4. Prognostication
  Fifteen studies explored the use of AI models for prognostication of head and neck cancers.^55^, ^56^, ^57^, ^58^, ^59^, ^60^, ^61^, ^62^, ^63^, ^64^, ^65^, ^66^, ^67^, ^68^, ^69^
  Six studies focused on PTC prognostication predicting central lymph node metastasis (CLNM) and recurrence, integrating imaging, time series thyroid function testing, clinicodemographic, and pathologic data.^55^, ^56^, ^65^, ^66^, ^67^, ^68^ These custom‐built multimodal AI architectures achieved AUCs ranging from 0.65 to 0.96, with some models outperforming radiologists. Under equal sensitivity or specificity, the models demonstrated a 6.5% and 2.9% increase in sensitivity and specificity, respectively, compared to the average radiologist's (specificity 0.85, sensitivity 0.51).^66^
  In oropharyngeal or nasopharyngeal malignancies, custom‐developed AI models trained on image, text, and genomic data demonstrated C‐indices from 0.52 to 0.79 and AUCs between 69% and 95% in their test or external validation cohorts for prognostic outcomes such as disease‐free or overall survival, distant metastasis, and locoregional recurrence.^57^, ^58^, ^59^, ^60^, ^61^, ^62^, ^63^, ^69^
  Similar to diagnostics, models integrating multimodal inputs performed better than single modality comparators within the study.^55^, ^56^, ^57^, ^58^, ^59^, ^60^, ^61^, ^62^, ^63^, ^64^, ^65^, ^66^, ^67^, ^68^, ^69^ For example, Han et al (2024) developed an integrated model to predict occult lymph node metastasis in early‐stage tongue cancer, combining Resnet50 for deep transfer learning on imaging data and clinical and radiomic features processed using a range of SVMs, random forests, and gradient boosting methods. This multimodal model outperformed junior physicians and demonstrated performance comparable to more senior physicians.^69^ However, when tested on external institutional data sets, the highest performing models observed significant reductions in performance.^61^ Clinical implementation requires further prospective validation but to enhance generalizability.
  Attention must also be given to the composition of data sets used in training prognostic algorithms. In certain head and neck malignancies, such as rhinology, patients from lower socioeconomic backgrounds and minority populations are more likely to present with advanced‐stage disease.^70^, ^71^, ^72^ If uncorrected, this distribution may inadvertently bias models to determine these individuals as disproportionally low risk for early‐stage malignancy, leading to inaccurate risk stratification and potentially inequitable treatment recommendations.^70^, ^71^, ^73^
  Incorporating supervised learning approaches may help mitigate such biases by enabling greater control over input features and training labels.^74^
  Beyond concerns related to biased training data, the use of identifiable health information in AI systems raises substantial patient privacy risks and may contravene existing regulatory frameworks. Although many studies rely on de‐identified data sets or publicly available registries, re‐identification remains possible through triangulation with external data sources with overlapping quasi‐identifiers such as demographics, location, or temporal identifiers. As the number of accessible data sets hosted by large‐scale information technology infrastructure grows, the ability to cross‐reference information across platforms can undermine de‐identification safeguards.^75^ Breaches of patient privacy may carry significant consequences, including workplace discrimination, insurance inflation, or psychological distress stemming from perceived loss of confidentiality.^76^ As such, real‐world clinical implementation will require institutionally governed data pipelines, secure deployment architectures, and robust regulatory oversight.
5. Other applications
  A smaller subset of studies investigated other applications. One study proposed a CNN‐based tool for automated sinus CT interpretation during the workup of CRS, achieving 100% segmentation success and strong correlation between the degree of sinus opacification with established clinical scoring systems such as Lund‐Mackay and Lund‐Kennedy.^77^ Another study built a nomogram, machine learning, and CNN models to predict post‐operative facial nerve outcomes in patients with acoustic neuroma using clinical data and raw MRI features, achieving an AUC of 0.89 and overall accuracy of 81%.^78^

An exploratory study demonstrated the capacity of generative multimodal AI to create visual representations of patient‐reported symptoms such as tinnitus and sinus pressure.^79^ Using text prompts and initial anatomical sketches, the model generated conceptual visualizations that translated subjective symptom experiences, such as the destabilizing perception associated with vertigo, through interpretable or character‐based imagery. While not yet clinically validated, this use suggests the potential for AI‐enhanced patient communication tools.

## Implications for Practice

Multimodal LLMs represent a rapidly advancing frontier in otolaryngology and are emerging as powerful tools with promising utility for diagnosis, risk stratification, treatment planning, and education. By integrating multiple data types, these systems consistently outperformed unimodal approaches in experimental settings. Their potential to enhance various aspects of otolaryngologic clinical practice, trainee education, and patient outcomes is significant. However, their beneficial integration must be balanced with ethical responsibility.

In light of limitations and ethical considerations identified across reviewed literature, the development and implementation of multimodal AI in otolaryngology may benefit from a principle‐based framework which considers: (1) robust data governance and privacy protections, including de‐identification, secure storage, and regulatory compliance; (2) mitigation of bias through representative data sets, external validation, and transparent reporting of demographic performance; (3) transparency and interpretability of model inputs and outputs to minimize reliance on opaque “black box” decisions; (4) clearly defined human oversight and accountability with escalation pathways for discordant outputs; and (5) continuous post‐deployment monitoring to detect performance drift and enable iterative improvement.

The infancy of this field renders most studies to remain in early stages, with retrospective designs, small sample sizes, and a lack of external, prospective, or multi‐institutional validation. There is also significant variability in the AI models used, ranging from open‐source frameworks (eg, BERT, LLaMA) to proprietary black box systems (eg, GPT‐4, Gemini), each with differing levels of transparency, reproducibility, and clinical adaptability.

Without formal guidelines for development, validation, and use, there remains substantial risk of diagnostic error, privacy breaches, and inappropriate reliance on AI‐generated outputs. Further, the training data raise questions for patient privacy and data security, involving unauthorized access and misuse of information. As the technology advances, it is imperative that regulatory frameworks and ethical standards evolve in parallel.

Additionally, the underrepresentation of marginalized groups in training data sets may exacerbate existing health disparities, particularly in early‐stage disease detection. Further compounding this is the under‐development of language optimization in LLMs. This is particularly so in low‐resource settings where linguistic and infrastructural barriers may hinder model performance and exaggerate inequities. Improving multilingual capabilities is essential for ensuring that LLMs are inclusive and universally applicable.

Clinicians should remain vigilant to the limitations of current models, including erroneous hallucinations and automation bias. LLMs should be helpful adjuncts, not replacements, for clinical judgment. Although some multimodal AI systems now demonstrate performance at a level comparable to junior trainees, their current capability lies primarily in augmenting decision‐making, improving consistency, and assisting with triage rather than operating independently. As with medical trainees, graduated supervision and physician oversight are expected to remain essential for high‐stakes diagnostic and therapeutic decisions, whereas lower‐risk administrative and workflow applications may utilize multimodal AI platforms.

Future research should prioritize prospective, multi‐institutional validation studies, bias mitigation strategies, and the development of inclusive, multilingual capabilities. Additionally, the underexplored role of LLMs in patient‐facing applications, such as improving health literacy, shared decision‐making, and communication across language barriers, represents a critical area for future investigation.

Given the rapid progression of technological advancement, this study may not reflect all recent developments in the field, particularly those published after our search date. However, this study provides a meaningful basis for otolaryngologists to understand and evaluate the role of LLMs in their current and future practice.

## Author Contributions

**Ying Jie Li,** conceptualization, methodology, formal analysis, investigation, data curation, writing—original draft, writing—review and editing; **Flora Su,** data curation, visualization, writing— review and editing; **Norbert Banyi,** conceptualization, data curation, writing—review and editing; **Philip Edgcumbe,** writing—review and editing; **Andrew Thamboo,** writing—review and editing; **Ameen Amanian,** supervision, writing—review and editings.

## Disclosures

### Competing interests

The authors declare no conflicts of interest.

### Funding source

None.

## Supporting information


**Supporting Information**.

**Supporting Information**.

**Supporting Information**.
