# Supplementary material for: Clinical Applications of Multimodal Artificial Intelligence in Otolaryngology: A State‐of‐the‐Art Review
Source: Otolaryngol Head Neck Surg. 2026 May 12;175(2):304–15. doi: 10.1002/ohn.70285 (PMC13418058; doi:10.1002/ohn.70285)
Supplement: Supplementary file 1 — Supporting Information. [file OHN-175-304-s003.docx]

**Supplemental Table S1**

*Search Strategy:*

**Medline (n= 2277):**

| **#** | **Query** |
| --- | --- |
| **1** | **exp Otolaryngology/** |
| **2** | **ENT.mp. [mp=title, book title, abstract, original title, name of substance word, subject heading word, floating sub-heading word, keyword heading word, organism supplementary concept word, protocol supplementary concept word, rare disease supplementary concept word, unique identifier, synonyms, population supplementary concept word, anatomy supplementary concept word]** |
| **3** | **("Ear, Nose and Throat" or laryngology or rhinology or otology).mp. [mp=title, book title, abstract, original title, name of substance word, subject heading word, floating sub-heading word, keyword heading word, organism supplementary concept word, protocol supplementary concept word, rare disease supplementary concept word, unique identifier, synonyms, population supplementary concept word, anatomy supplementary concept word]** |
| **4** | **1 or 2 or 3** |
| **5** | **exp "Head and Neck Neoplasms"/** |
| **6** | **("head and neck" adj2 (neoplasm* or cancer* or surger* or oncology)).mp. [mp=title, book title, abstract, original title, name of substance word, subject heading word, floating sub-heading word, keyword heading word, organism supplementary concept word, protocol supplementary concept word, rare disease supplementary concept word, unique identifier, synonyms, population supplementary concept word, anatomy supplementary concept word]** |
| **7** | **exp Otorhinolaryngologic Diseases/** |
| **8** | **(otolaryngolog* or Otorhinolaryngolog*).mp. [mp=title, book title, abstract, original title, name of substance word, subject heading word, floating sub-heading word, keyword heading word, organism supplementary concept word, protocol supplementary concept word, rare disease supplementary concept word, unique identifier, synonyms, population supplementary concept word, anatomy supplementary concept word]** |
| **9** | **exp Deep Learning/ or exp natural language processing/** |
| **10** | **("Vision-language model*" or "Image-text model*" or "Speech-text model*" or "Audio visual model*" or ViLBERT or VisualBERT or "Vision and Language BERT" or DALL-E or DALL-E2 or VQA or "Visual Question Answering" or PaLM-E or LLaVA or "large language and vision assistant").mp. [mp=title, book title, abstract, original title, name of substance word, subject heading word, floating sub-heading word, keyword heading word, organism supplementary concept word, protocol supplementary concept word, rare disease supplementary concept word, unique identifier, synonyms, population supplementary concept word, anatomy supplementary concept word]** |
| **11** | **(multimodal adj1 ("artificial intelligence" or AI or GPT or "deep learning" or "sensor fusion")).mp. [mp=title, book title, abstract, original title, name of substance word, subject heading word, floating sub-heading word, keyword heading word, organism supplementary concept word, protocol supplementary concept word, rare disease supplementary concept word, unique identifier, synonyms, population supplementary concept word, anatomy supplementary concept word]** |
| **12** | **(GPT-4* or GPT-4V or GPT-4 Vision or VisualGPT or GPT-4o).mp. [mp=title, book title, abstract, original title, name of substance word, subject heading word, floating sub-heading word, keyword heading word, organism supplementary concept word, protocol supplementary concept word, rare disease supplementary concept word, unique identifier, synonyms, population supplementary concept word, anatomy supplementary concept word]** |
| **13** | **("cross-modal" adj2 (AI or "artificial intelligence" or "deep learning" or GPT)).mp. [mp=title, book title, abstract, original title, name of substance word, subject heading word, floating sub-heading word, keyword heading word, organism supplementary concept word, protocol supplementary concept word, rare disease supplementary concept word, unique identifier, synonyms, population supplementary concept word, anatomy supplementary concept word]** |
| **14** | **contrastive language-image pre-training.mp. [mp=title, book title, abstract, original title, name of substance word, subject heading word, floating sub-heading word, keyword heading word, organism supplementary concept word, protocol supplementary concept word, rare disease supplementary concept word, unique identifier, synonyms, population supplementary concept word, anatomy supplementary concept word]** |
| **15** | **bootstrapping language-image pre-training.mp. [mp=title, book title, abstract, original title, name of substance word, subject heading word, floating sub-heading word, keyword heading word, organism supplementary concept word, protocol supplementary concept word, rare disease supplementary concept word, unique identifier, synonyms, population supplementary concept word, anatomy supplementary concept word]** |
| **16** | **("large language model*" or "transformer model*" or "generative pre-trained transformer" or "generative AI").mp. [mp=title, book title, abstract, original title, name of substance word, subject heading word, floating sub-heading word, keyword heading word, organism supplementary concept word, protocol supplementary concept word, rare disease supplementary concept word, unique identifier, synonyms, population supplementary concept word, anatomy supplementary concept word]** |
| **17** | **9 or 10 or 11 or 12 or 13 or 14 or 15 or 16** |
| **18** | **4 or 5 or 6 or 7 or 8** |
| **19** | **17 and 18** |

**Embase (n= 593)**

**Web of Science (n=720)**

**Scopus (n=240)**

**Cochrane Central Register of Controlled Trials (n= 223)**

**CINAHL (N-34)**
