# Supplementary material for: Clinical Applications of Multimodal Artificial Intelligence in Otolaryngology: A State‐of‐the‐Art Review
Source: Otolaryngol Head Neck Surg. 2026 May 12;175(2):304–15. doi: 10.1002/ohn.70285 (PMC13418058; doi:10.1002/ohn.70285)
Supplement: Supplementary file 2 — Supporting Information. [file OHN-175-304-s001.docx]

**Supplemental Table S2.** Summary of study characteristics. Google Bard has been rebranded as Gemini, while Microsoft Bing Chat is now known as Copilot

| **Study ID** | **Study Design** | **Subspecialty** | **Country of Senior Author** | **Application** | **Specific Application** | **AI model used** | **Level of Evidence** |
| --- | --- | --- | --- | --- | --- | --- | --- |
| Lee 2024 | Performance Evaluation | Head & Neck Oncology | Korea | Clinical | Prognostication | Custom architecture: Multilayer perceptron block, long short-term memory block, ensemble block | NA |
| Wang 2024 | Performance Evaluation | Head & Neck Oncology | China | Clinical | Prognostication | Custom architecture: Image feature extraction, text feature extraction, and feature fusion module | NA |
| Liang 2025 | Performance Evaluation | Head & Neck Oncology | China | Clinical | Diagnosis and Disease Classification | Commercial: GPT-4o, Claude 3.5 Sonnet, Gemini 1.5 Pro Open-Source: LLaVA-Med, InternVL, HuatuoGPT-Vision | NA |
| Ding 2024 | Performance Evaluation | General ENT | China | Clinical | Diagnosis and Disease Classification | ChatGPT-4 | NA |
| Mohseni 2024 | Performance Evaluation | Head & Neck Oncology | USA | Clinical | Diagnosis and Disease Classification | ChatGPT | NA |
| Zhang 2024 | Performance Evaluation | Head & Neck Oncology | China | Clinical | Treatment planning and evaluation | Custom architecture: 3D convolutional autoencoder 3-step feature selection method (statistical tests, Least Absolute Shrinkage and Selection Operator, and Recursive Feature Elimination), random forest EF | NA |
| Rajendran 2025 | Performance Evaluation | Head & Neck Oncology | USA | Clinical | Treatment planning and evaluation | Custom architecture: Medformer Commercial: GPT-4 | NA |
| Liang 2025 | Performance Evaluation | Head & Neck Oncology | China | Clinical | Treatment planning and evaluation | Custom architecture: 3DResNet, XGBoost | NA |
| Qi 2025 | Performance Evaluation | Head & Neck Oncology | China | Clinical | Treatment planning and evaluation | Custom architecture: ResNet-50, SVM | NA |
| Miao 2025 | Performance Evaluation | Head & Neck Oncology | China | Clinical | Prognostication | Custom architecture: ResNet-50 | NA |
| Pradhan 2025 | Performance Evaluation | Head & Neck Oncology | India | Clinical | Diagnosis and Disease Classification | ChatGPT-3.5, ChatGPT-4.0, ChatGPT-4o, Gemini | NA |
| Wang 2025 | Performance Evaluation | Head & Neck Oncology | China | Clinical | Treatment planning and evaluation | Self- developed: VGG16, XGBoost, Logistic Regression, SVM, KNN | NA |
| Rajendran 2025 | Performance Evaluation | Head & Neck Oncology | USA | Clinical | Treatment planning and evaluation | Custom architecture: Radformer Commercial: ChatGPT-4 | NA |
| Chen 2024 | Performance Evaluation | Head & Neck Oncology | China | Clinical | Diagnosis and Disease Classification | ChatGPT-4o, Claude 3-Opus | NA |
| Mete 2024 | Performance Evaluation | General ENT | Turkey | Clinical | Medical Education | ChatGPT-4, Gemini, Copilot | NA |
| Javan 2024 | Performance Evaluation | General ENT | USA | Clinical | Patient Communication | Midjourney, DALL-E3, Kaiber.ai | NA |
| Yao 2022 | Performance Evaluation | Head & Neck Oncology | China | Clinical | Prognostication | Custom architecture: DeepThy-Net, ResNet-50, DenseNet121, InceptionV4, CSAC-Net | NA |
| Chen 2023 | Performance Evaluation | Head & Neck Oncology | China | Clinical | Diagnosis and Disease Classification | Custom architecture: Xception, CBAM attention module, BERT | NA |
| Woo 2023 | Performance Evaluation | Head & Neck Oncology | Korea | Clinical | Prognostication | Custom architecture: Random Forest, LightGBM, ExtraTrees, XGBoost, AdaBoost, logistic regression (LR) | NA |
| Quan 2024 | Performance Evaluation | Head & Neck Oncology | China | Clinical | Treatment planning and evaluation | Custom architecture: XGBoost | NA |
| Yu 2024 | Performance Evaluation | Head & Neck Oncology | China | Clinical | Prognostication | Custom architecture: ResNet-50, CLAM, attention gate module | NA |
| Zhong 2020 | Performance Evaluation | Head & Neck Oncology | China | Clinical | Prognostication | Custom architecture: SE-ResNeXt | NA |
| Qiang 2021 | Performance Evaluation | Head & Neck Oncology | China | Clinical | Prognostication | Custom architecture: XGBoost | NA |
| Le 2022 | Performance Evaluation | Head & Neck Oncology | Canada | Clinical | Prognostication | Custom architecture: PreSANet,CNN, global context block, random forest, logistic regression | NA |
| Dohopolski 2022 | Performance Evaluation | Head & Neck Oncology | USA | Clinical | Treatment planning and evaluation | Custom architecture: Logistic regression, SVM, multilayer perception, ResNet50, MedicalNet | NA |
| Zhao 2022 | Performance Evaluation | Head & Neck Oncology | China | Clinical | Prognostication | Custom architecture: EfficientNet-lite0, MobileNet-V2, MobileNet-V3, DenseNet121, ResNet18, integrated nomogram | NA |
| Massey 2022 | Performance Evaluation | Rhinology | USA | Clinical | Diagnosis and Disease Classification | Custom architecture: Densely Connected Convolutional Networks based on the Tiramisu method | NA |
| Wang 2023 | Performance Evaluation | Neurotology | China | Clinical | Post-operative Outcome Prediction | Custom architecture: SVM, kNN, light gradient boosting machine, Decision tree, random forest, extra trees, logistic regression | NA |
| Patel 2024 | Performance Evaluation | Rhinology | USA | Clinical | Medical Education | ChatGPT-3.5, ChatGPT-4 | NA |
| Noda 2024 | Performance Evaluation | Otology | Japan | Clinical | Diagnosis and Disease Classification | ChatGPT-4Vision | NA |
| Liu 2024 | Performance Evaluation | Head & Neck Oncology | USA | Clinical | Treatment planning and evaluation | Custom architecture: GPT-RadPlan Commercial: GPT-4Vision | NA |
| Lu 2023 | Performance Evaluation | Head & Neck Oncology | China | Clinical | Treatment planning and evaluation | Custom architecture: Multi-modal MRI feature fusion block, variational autoencoder module, multi scale feature fusion network, adaptive task fature fusion, random forest, global max pooling | NA |
| Kazmierski 2023 | Performance Evaluation | Head & Neck Oncology | Canada | Clinical | Prognostication | Custom architecture: 12 crowd-sourced models developed by independent investigators. Including: Deep multitask LR, fuzzy LR and cox proportional hazards, 3D convnet, 2D convnet, 3D DenseNet, multilayer perceptron | NA |
| Lin 2024 | Performance Evaluation | Head & Neck Oncology | USA | Clinical | Diagnosis and Disease Classification | Custom architecture: Random Forest classifier | NA |
| Vollmer 2024 | Performance Evaluation | Head & Neck Oncology | Germany | Clinical | Prognostication | Custom architecture: Random survival forest, gradient boosting survival analysis, fast survival SVM, DeepSurv | NA |
| Lu 2024 | Performance Evaluation | Neurotology | China | Clinical | Diagnosis and Disease Classification | Custom architecture: Temporal difference module, big-kernel long-term module | NA |
| Sievert 2024 | Performance Evaluation | Head & Neck Oncology | Germany | Clinical | Diagnosis and Disease Classification | ChatGPT-4Vision | NA |
| Wang 2024 | Performance Evaluation | Head & Neck Oncology | China | Clinical | Prognostication | Custom architecture: ResNet | NA |
| Noda 2024 | Performance Evaluation | General ENT | Japan | Clinical | Medical Education | ChatGPT-4Vision | NA |
| Han 2024 | Performance Evaluation | Head & Neck Oncology | China | Clinical | Prognostication | Custom architecture: ResNet-50, SVM, kNN, random forest, extra trees, XGBoost, light gradient boosting machine, logistic regression | NA |
| Ma 2024 | Performance Evaluation | Head & Neck Oncology | Netherlands | Clinical | Prognostication | Custom architecture: TransRP, DenseNet121, DeiT-S, DeepSurv, ResNet18 | NA |
| Rajendran 2024 | Performance Evaluation | Head & Neck Oncology | USA | Clinical | Treatment planning and evaluation | Custom architecture: Medformer, GPT-4, Pubmed BERT | NA |
| Terwilliger 2024 | Performance Evaluation | General ENT | USA | Clinical | Medical Education | ChatGPT-4, Gemini | NA |
| Maniaci 2024 | Performance Evaluation | Laryngology | France | Clinical | Diagnosis and Disease Classification | ChatGPT-4 | NA |
