# Supplementary material for: Clinical Applications of Multimodal Artificial Intelligence in Otolaryngology: A State‐of‐the‐Art Review
Source: Otolaryngol Head Neck Surg. 2026 May 12;175(2):304–15. doi: 10.1002/ohn.70285 (PMC13418058; doi:10.1002/ohn.70285)
Supplement: Supplementary file 3 — Supporting Information. [file OHN-175-304-s002.docx]

**Supplemental Table S3**

| **Study ID** | **AI model used** | **Input modalities** | **Sample Size** | **Methodology** | **Outcomes** |
| --- | --- | --- | --- | --- | --- |
| Lee 2024 | Custom architecture: Multilayer perceptron block, long short-term memory block, ensemble block | Text, Time-series | 1613 patients (1550 non-recurrent, 63 recurrent) | Clinical information and time series data (e.g. post-operative thyroid function test results) were used to develop a model to predict the recurrence of PTC after thyroidectomy | The LMM achieved an AUC of 0.9622, F1-score of 0.4603, sensitivity of 0.9042, and specificity of 0.9077 |
| Wang 2024 | Custom architecture: Image feature extraction, text feature extraction, and feature fusion module | Image, text | 105 patients | MRI images and preoperative clinicopathologic data from PTC patients were used to develop a LMM to predict central lymph node metastasis in PTC. | The combined model achieved AUC at 0.863. |
| Liang 2025 | Commercial: GPT-4o, Claude 3.5 Sonnet, Gemini 1.5 Pro Open-Source: LLaVA-Med, InternVL, HuatuoGPT-Vision | Image, text | 50 patients, 169 images, 1084 questions | Image and text prompts were provided to the multimodal large language models and asked for the most likely category of diagnosis. | Commercial models achieved accuracy ranging from 67.53% - 79.43%, outperforming open source models (accuracy 41.14% - 60.52%). There was a 19% performance gap difference between the best-performing commercial model (Claude 3.5 Sonnet) and the top open-source model (HuatuoGPT-Vision). |
| Ding 2024 | ChatGPT-4 | Image, Text | 44 histopath images from 16 organs | ChatGPT-4 was presented with normal and pathological images and asked to identify the organ or presence and type of tumour. | GPT-4 achieved an overall accuracy of 64% in identifying tumor imaging and tissue origins. |
| Mohseni 2024 | ChatGPT | Image, Text | 200 histopathological cases | ChatGPT was prompted to generate a differential diagnosis from provided clinical summaries and pathology images. | ChatGPT successfully included the correct diagnosis in 100 % of cases (200/200). The correct diagnosis was ranked 1st in 49.5 % of cases. |
| Zhang 2024 | Custom architecture: 3D convolutional autoencoder 3-step feature selection method (statistical tests, Least Absolute Shrinkage and Selection Operator, and Recursive Feature Elimination), random forest EF | Radiomics, text | 175 retrospective, 27 prospective patients | Radiomics from CT images and clinical information were provided to a Custom architecture model to predict the occurence of esophageal fistula in radiotherapy or chemotherapy patients. | The model achieved AUC between 0.81 - 0.89 in training, test, and prospective cohorts. It's predictive validity was validated by Brier-score and calibration curve analyses. |
| Rajendran 2025 | Custom architecture: Medformer Commercial: GPT-4 | Image, text | 668 patients (535 public oropharyngeal carcinoma data + 133 in-house prostate cancer data) | A visual language attention module (Medformer) was developed to assist in target volume delineation for radiation therapy. | On the oropharyngeal carcinoma dataset, Medformer achieved a DSC of 0.77 ± 0.11 , IOU of 0.70 ± 0.09, and HD95 of 7.52 ± 4.8 mm. For delineating the clinical target volume, Medformer achieved a DSC of 0.91 ± 0.04, IOU of 0.85 ± 0.05, and HD95 of 2.98 ± 1.60 mm, comparable with other state-of-the-art algorithms. |
| Liang 2025 | Custom architecture: 3DResNet, XGBoost | Image, text | 999 patients | A model was developed to extract deep learning MRI features and integrate them with clinical information to stratify patients into high- and low-risk groups to detmine whether intensity modulated radiotherapy (IMRT) alone was sufficient for treatment of stage II nasopharyngeal carcinoma. | The combined model achieved a concordance index values ranging from 0.768 - 0.804 across training, internal validation, and external test cohorts. Compared to those who recieved additional chemotherapy, low risk patients treated with IMRT alone had comparable or superior progression-free survival. |
| Qi 2025 | Custom architecture: ResNet-50, SVM | Radiomics, pathomics, text | 223 patients | Radiomics from contrast-enhanced CT images, pathomics from whole-slide images of pathological specimens, and clinicopathological features were combined to construct a model used to predict complete response to neoadjuvant chemoimmunotherapy in locally advanced esophageal cancer | The multi-modality model yielded an area under the curve (AUC) of 0.89 (95% confidence interval [CI], 0.75-1.00), outperforming the unimodal models (AUC 0.63 - 0.77). |
| Miao 2025 | Custom architecture: ResNet-50 | Image, radiomics, text | 661 patients | A multi-modal multiregion nomogram (MMRN) was developed using US images, CT images, and fat radiomics to predict central lymph node metastasis in papillary thyroid carcinoma. Results were compared with five radiologists. | The model achieved AUCs between 0.818 and 0.829 across internal and external test sets. Calibration curves indicated good agreement between predicted and actual outcomes, and decision curve analysis supported clinical utility. Compared to radiologists, the model improved performance by up to 6.5% in specificity-matched or 2.9% in sensitivity-matched scenarios. |
| Pradhan 2025 | ChatGPT-3.5, ChatGPT-4.0, ChatGPT-4o, Gemini | Image, Text | 42 case reports | Case reports and images representing potentially malignant oral lesions were presented to GPT 3.5, 4.0 (prompted with text only) and Gemini and GPT-4o (prompted with text and images) for diagnosis. Responses were reviewed by 2 subject matter experts. | GPT 4o got the maximum number of correct responses (27/42), followed by GPT 4.0 (20/42), GPT 3.5 (18/42) and Gemini (15/42). GPT 4o aligns most closely with the subject experts in diganostic accuracy (Œ = 0.514 - 0.659) and made incorrect recognitions in 6 out of 42 cases (14.2%). In comparison, Gemini incorrectly identified 14 out of 42 cases (33.3%) and responded significantly different compared to subject experts (Œ = 0.245 - 0.326, p<0.05). |
| Wang 2025 | Self- developed: VGG16, XGBoost, Logistic Regression, SVM, KNN | Image, text, radiomics | 337 patients, 2 hospital sites | Radiomics, deep learning features from multi-sequence MRI images, and clinical data from NPC patients undergoing radiotherapy to predict treatment efficacy. | The manual radiomics model based on XGBoost and the deep learning model based on KNN (the AUCs in the training set: 0.909, 0.823, respectively) showed better predictive efficacy than other machine learning algorithms. The stacked model that integrated MRI-based deep learning radiomics, fMRI, and hematological indicators, has the strongest efficacy prediction ability of AUC in the training set [0.984 (95%CI: 0.972‚Äì0.996)], the internal validation set [0.936 (95%CI: 0.885‚Äì0.987)], and the external validation set [0.959 (95%CI: 0.901‚Äì1.000)]. |
| Rajendran 2025 | Custom architecture: Radformer Commercial: ChatGPT-4 | Image, text | 2985 patients | Clinical data and imaging was combined to create a model to assist in automation of radiation therapy target volume delineation in head-and-neck cancer patients. | The Radformer demonstrated superior performance in segmenting RT target volumes compared to state-of-the-art models. On the head-and-neck cancer dataset, Radformer achieved a mean DSC of 0.76 ¬± 0.09 versus 0.66 ¬± 0.09, a mean IOU of 0.69 ¬± 0.08 versus 0.59 ¬± 0.07, and a mean HD95 of 7.82 ¬± 6.87 mm versus 14.28 ¬± 6.85 mm for gross tumor volume delineation, compared to the baseline 3D-UNETR. |
| Chen 2024 | ChatGPT-4o, Claude 3-Opus | Text, Image | 112 patients, 116 thyroid nodules (75 benign, 41 malignant) | ChatGPT-4o and Claude 3-Opus were presented with ultrasound images and clinical information to diagnose thyroid noduls as benign or malignant. | ChatGPT-4o and Claude achieved AUC of 57.0% and 52.0% respectively, with poor agreement with pathological results (kappa 0.034 - 0.116) and high rates of unnecessary biopsy (41.4% - 43.1%; whereas a junior radiologist achieved AUC of 72.4% and 12.1% rate of unnecessary biopsy |
| Mete 2024 | ChatGPT-4, Gemini, Copilot | Text, Image | 22 participants, 316 questions | Accuracy of LMM performance on questions from the Resident Training Textbook of the Turkish Society of Otorhinolaryngology Head and Neck Surgery was assessed and compared against residents. | Accuracy for LMMs are as follows: GPT-4 54.75%, Gemini 40.50% and Bing 37.00%. Their performance were comparable to junoir residents (accuracy 46.90%) but could not compare to senior residents (accuracy 75.5%, p<0.001). |
| Javan 2024 | Midjourney, DALL-E3, Kaiber.ai | Text, Image | NA | Text prompts and initial sketches (e.g. of the ossicles) was provided to LMMs to generate images illustrating clinical symptoms experienced by otolaryngologist patients. | The LMM generated outer, middle, inner ear images, head and neck symptoms e.g. tinnitus, migraine, sinus pressure, vertigo, sea sickness, mal de debarquement, Dysgeusia, xerostomia, odynophagia, dizziness, bloodshot eyes with thunderclap headache, and seasonal allergies. |
| Yao 2022 | Custom architecture: DeepThy-Net, ResNet-50, DenseNet121, InceptionV4, CSAC-Net | image, text | 6032 patients (23617 ultrasound images), 3 hospital sites | Ultrasound images and clinical factors of PTC patients was used to develop a model to predict different CLNM patterns | The model achieved AUC between 0.870 - 0.905. |
| Chen 2023 | Custom architecture: Xception, CBAM attention module, BERT | Text, image | 323 patients | Ultrasound images and clinical data were presented to a LMM to predict the risk of follicular thyroid carcinoma | The LMM demonstrated AUC of 0.97, achieving better performance than using grey scale ultrasound images, color doppler ultrasound images, and patient clinical data alone. |
| Woo 2023 | Custom architecture: Random forest, LightGBM, ExtraTrees, XGBoost, AdaBoost, logistic regression (LR) | image, text | 126 patients, 19 external test from 2 other hospitals | Models was developed to predict HPV status in oropharyngeal squamous cell carcinoma patients using either 18F-FDG PET-computed tomography scans, clinical parameters, or both. Their predictive performance was compared. | The model combining both modalities achieved the best performance (AUC = 0.78), outperforming both unimodal models |
| Quan 2024 | Custom architecture: XGBoost | text, radiomics, omics | 219 patients | A model was construted using clinical features, DVH features, radiomics and dosiomics featuresto predict tadiation induced hypothyroidism in nasopharyngeal carcinoma patients undergoing chemotherapy. | The combined model achived AUC 0.842, outperforming all models provided with only one input (AUC 0.673 - 0.798) |
| Yu 2024 | Custom architecture: ResNet-50, CLAM, attention gate module | images, omics | 1011 patients (institutional data + The Cancer Genome Atlas) | Matched histopathology images, genomic, transcriptomic, and immune cell data were combined to construct a LMM to predict lymph node metastasis and disease-free survival (DFS) in PTC | The model achieved AUC of 0.83 - 0.86 in the training, validation and real-world cohorts. |
| Zhong 2020 | Custom architecture: SE-ResNeXt | Radiomics, text | 638 patients | MRI scans of nasopharyngeal carcinoma patients recieving induction chemotherapy were taken prior to the addition of concurrent radiotherapy. Radiomics were combined with clinical prognostic parameters to build a model used to predict disease-free survival. | The integrated model significantly improved the predictive value compared to the clinical model alone (C-index: 0.771-0.788 vs. 0.625 - 0.640, p < 0.001). |
| Qiang 2021 | Custom architecture: XGBoost | text, image | 3444 patients, 4 hospitals | Clinical data and MRI images of locoregionally advanced nasopharyngeal carcinoma patients were used to build a model tasked to risk stratify patients to determine whether induction chemotherapy was needed in addition to concurrent chemoradiotherapy. | The model demonstrated a concordance index of 0.746- 0.776 in the internal and external validation cohorts, statistically outperforming the conventional TNM staging system |
| Le 2022 | Custom architecture: PreSANet,CNN, global context block, random forest, logistic regression | Text, Image | 669 patients, 4 institutions | A LMM was developed for the prediction of distant metastasis, locoregional recurrence, and overall survival occurrence probabilities within the 10 year follow-up time of head and neck squamous cell carcinoma patients | The model achieved a mean accuracy for distant metastasis, locoregional recurrence and overall survival of s 72%, 70% and 71%, respectively |
| Dohopolski 2022 | Custom architecture: Logistic regression, SVM, multilayer perception, ResNet50, MedicalNet | Image, omics, text | 271 patients | Imaging, dosimetric, and clinical data were ensembled tocreate a LMM for the early prediction of feeding tube placement in head and neck radiotherapy patients | The LMM achieved an AUC of 0.75, and statistically outperformed the clinical model alone (p = 0.001). |
| Zhao 2022 | Custom architecture: EfficientNet-lite0, MobileNet-V2, MobileNet-V3, DenseNet121, ResNet18, integrated nomogram | image, text | 420 patients | Clinical factors and PET/CT signatures were combined to create a LMM to predict survival in recurrent NPC patients. Performance was compared to single-modality models. | The C-index of the LMM was 0.732 - 0.741, significantly outperforming single modality approaches. The model effectively stratified patients into high- and low-risk groups, with hazard ratios above 4 in both training and test sets. Risk prediction remained consistent across subgroups defined by sex, N-stage, and EBV DNA levels. |
| Massey 2022 | Custom architecture: Densely Connected Convolutional Networks based on the Tiramisu method | Text, Image | 88 patients | CT images, serum eosinophil count, surgical pathology specimens, and clinical data were used to develop the algorithm for sinus cavity segmentation and disease quantification via %sinus opacification. | 100% of scans from various scanners were successfully segmented by the model. Percentage opcification demonstrated strong to moderate correlations to the Lund-Mackay score, Lund-Kennedy score; and percentage osteitis was moderately correlated with the Global Osteitis Scoring Scale. |
| Wang 2023 | Custom architecture: SVM, kNN, light gradient boosting machine, Decision tree, random forest, extra trees, logistic regression | text, radiomics | 110 patients | Clinical and radiomic features from MRI images were combined to construct a model to predict the prognosis of facial nerve function following surgery for acoustic neuroma | The comnimed model achived AUC 0.89 (95% CI, 0.84 - 0.91), outperforming all other developed models which used unimodal input. |
| Patel 2024 | ChatGPT-3.5, ChatGPT-4 | Text, image | 127 questions (93 text-based, 34 image-based) | ChatGPT 3.5 and GPT4 were presented with standardized rhinology questions from www.boardvitals.com and assess for performance as compared to average results of the question bank. | For text-based questions, ChatGPT 4 outperformed ChatGPT 3.5 (accuracy 45.2% v 86.0%, P = .0001). For image based questions, ChatGPT4 had an accuracy of 64.7%. |
| Noda 2024 | ChatGPT-4Vision | Text, Image | 305 images +190 test | Otoscopic images of middle ear diseases (acute otitis media, middle ear cholesteatoma, chronic otitis media, and otitis media with effusion) were combined with patient data and presented to GPT-4Vision to evaluate its diagnostic accuracy of middle ear diseases. Its performance was compared against 30 clinicians. | ChatGPT-4V achieved an accuracy of 82.1%, outperforming certified pediatricians (70.6%), but lacking from otolaryngologists (95%+). Disease-specific accuracy rates ranged from 76.5% - 89.19%. |
| Liu 2024 | Custom architecture: GPT-RadPlan Commercial: GPT-4Vision | Image, omics, text | 17 patients (6 head and neck cancer patients + 11 prostate cancer patients) | A model integrating radiation oncology knowledge with GPT-4Vision was built to assess and refine radiotherapy treatment plans (e.g. adjusting planning parameters, such as weights and objective doses) given dose distributions and dose-volume histograms. | Of the head & neck cases, the model provided better plans in 75%, reducing the mean dose by a 10-15% for most of the organs at risk. |
| Lu 2023 | Custom architecture: Multi-modal MRI feature fusion block, variational autoencoder module, multi scale feature fusion network, adaptive task fature fusion, random forest, global max pooling | Image, omics | 230 patients | MRI and dose-omics were used to build a LMM for the prediction of re-irradiation necrosis in recurrent nasopharyngeal carcinoma. | The model achieved AUC of 0.936, outperforming the single-modal model (AUC 0.78). |
| Kazmierski 2023 | Custom architecture: 12 crowd-sourced models developed by independent investigators | Text, Image, Radiomics | 2552 patients (internal) + 873 patients (external cohort) | Clinical data and pre-treatment CT images were presented to various LMMs to predict overall survival in head and neck cancer. | The most accurate model was trained with clinical data and tumor volume, outperforming models based solely on clinical features, radiomics, or complex deep networks for predicting 2-year and lifetime survival. However, its performance dropped notably when applied to external datasets, underscoring the need for population-specific reporting and robust validation. |
| Lin 2024 | Custom architecture: Random forest classifier | Text, Image | 18 patients | A LMM was tasked to classify malignant from benign when given imaging and perioperative clinical data from thyroid adenoma and carcinoma patients. | The LMM achieved AUROC of 0.79 (95% CI: 52%-100%), demonstrating higher sensitivity, NPV, and PPV than both the clinical-only and image-only models. |
| Vollmer 2024 | Custom architecture: Random survival forest, gradient boosting survival analysis, fast survival SVM, DeepSurv | Omics, images, text | 406 patients | A LMM was created integrating clinical, pathological, or genetic features for the survival prediction of oral squamous cell carcinoma. | The LMM demonstrated c-index values from 0.515 - 0.722, outperforming all unimodal models across all methods. |
| Lu 2024 | Custom architecture: Temporal difference module, big-kernel long-term module | Video, vector | 518 patients | Eye movement videos and diagnostic data from patients with BPPV were collected and presented to a Custom architecture LMM for the diagnosis of BPPV. | The model attained an accuracy of 81.7%, precision 82.1%, sensitivity 94.1%, and specificity 96.5%. |
| Sievert 2024 | ChatGPT-4Vision | Image, Text | 5 participants, 155 CLE images | Chat-GPT-4V was presented with confocal laser endomicroscopy images of squamous cell carinoma (confirmed by histology) or healthy mucosa. Its performance was compared to surgeons and pathologists. | Chat GPT-4V achieved 71.2% accuracy, 85.1% (95% CI 79.9 - 89.2%) sensitivity, and 50.6% (95% CI 42.8 - 58.4%) specificity. Subject experts achieved an accuracy of 88.5%. |
| Wang 2024 | Custom architecture: ResNet | Image, text | 611 patients | Given preoperative ultrasound images and clinical data, the LMM was tasked to predict central lymph node metastasis preoperatively. | The model achieved an average AUC of 0.65 |
| Noda 2024 | ChatGPT-4Vision | Text, Image | 100 questions (54 text, 46 image) | ChatGPT-4 Vision's accuracy on questions from the 2023 otolaryngology board certification examination was assessed. | Text-only input yielded low accuracy (24.7%), which improved significantly with English translations (47.3%, P<.001). Accuracy was generally lower for image-based questions, which also had high nonresponse rates. Anatomy-related questions had the highest accuracy overall. For image-based questions, accuracy improved from 30.4% with text-only input to 41.3% when images were included (P=0.02). |
| Han 2024 | Custom architecture: ResNet-50, SVM, kNN, random forest, extra trees, XGBoost, light gradient boosting machine, logistic regression | Text, Radiomics | 125 patients | Preoperative contrast-enhanced CT and clinical data in tongue cancer patients were collected to develop a LMM for the prediction of occult lymph node metastasis. | The model achieved predictive accuracy of 84%, sensitivity of 100%, specificity of 76.5%, and an AUCROC of 0.949 (95% CI [0.870 - 1.000]). |
| Ma 2024 | Custom architecture: TransRP, DenseNet121, DeiT-S, DeepSurv, ResNet18 | Text, Image, Radiomics | 400 patients | Pretreatment PET/CT images and clinical data was presented to a self developed LMM to predict prognostic outcomes (locoregional control, distant metastasis-free survival, overall survival) for oropharyngeal squamous cell carcinoma patients | The model acheived C-indices of 0.61, 0.84 and 0.70 for locoregional control, distant metastasis-free survival, and overall survival, respectively. |
| Rajendran 2024 | Custom architecture: Medformer, GPT-4, Pubmed BERT | Text, Image | 2985 patients | CT images and clinical data were presented to a self developed model ("Radformer") and tasked to assist in delineation of treatment target volume in radiation therapy. | Radformer achieved a mean DSC of 0.76, a mean IOU of 0.69, and a mean HD95 of 7.82 mm, significantly outperforming other existing models across all outcome measures (p < 0.05). |
| Terwilliger 2024 | ChatGPT-4, Gemini | Image, text | 1077 text-only questions, 60 image + text questions | Questions from the otolaryngology board exam preparation tool BoardVitals were inputted into ChatGPT, GPT-4, and Google Bard to assess their response accuracy. | GPT-4 had the best overall image interpretation ability and scored significantly better than ChatGPT and Bard across all difficulty levels (78.7% vs 55.3% and 61.7% respectively, p<0.001). Easy questions yield the best performance from all models (accurary 69.7 % - 92.5%) compared to performance on hard questions (accuracy 42.3% - 61.3%). |
| Maniaci 2024 | ChatGPT-4 | Text, Image | 40 patients | ChatGPT-4 was presented with patient history and clinical videolaryngostroboscopic images and asked for differential diagnoses, management, and treatment(s). A 5-point Likert scale was used to assess case complexity. The responses were assessed by laryngologists. | ChatGPT-4 acheived a correct primary diagnosis in 20.0% - 25.0% of cases, a mean accuracy of 27.5% for image analysis, and high consistency with practitioners in 5 cases (12.5%; 4/5). However, ChatGPT-4 ordered extraneous examinations in 90 more cases than practitioners did and erroneously documented vocal fold irregularity (mass or lesion), glottic insufficiency, and vocal cord paralysis in 21 (52.5%), 2 (0.05%), and 5 (12.5%) cases, respectively. |
